# Effects of different cultivation conditions on the production of β-cyclocitral and β-ionone in *Microcystis aeruginosa*

**DOI:** 10.1186/s12866-022-02473-6

**Published:** 2022-03-24

**Authors:** Jéssica Aparecida Silva Moretto, Paloma Nathane Nunes de Freitas, Éryka Costa de Almeida, Lucas Miguel Altarugio, Simone Vieira da Silva, Marli de Fátima Fiore, Ernani Pinto

**Affiliations:** 1 Centre for Nuclear Energy in Agriculture, University of São Paulo, Piracicaba, SP Brazil; 2 Luiz de Queiroz College of Agriculture, University of São Paulo, Piracicaba, SP Brazil; 3 Faculty of Pharmaceutical Sciences, University of São Paulo, São Paulo, SP Brazil; 4 Food Research Center (FoRC – CEPID), University of São Paulo, São Paulo, SP Brazil

**Keywords:** Cyanobacteria, SPME, GC–MS, β-ionone, β-cyclocitral

## Abstract

**Background:**

Cyanobacteria blooms have become a major environmental problem and concern because of secondary metabolites produced by cyanobacteria released into the water. Cyanobacteria produce volatile organic compounds (VOCs), such as the compounds β-cyclocitral and β-ionone, which comprise odors, off-flavors, defense compounds, as well as growth regulators. Therefore, the general objective of this work was to evaluate the VOCs produced by two strains of *Microcystis aeruginosa*, differing in their ability to produce microcystins (LTPNA 01—non-producing and LTPNA 08—toxin-producing). The analysis of VOC production was carried out in (1) normal culture conditions, (2) under different light intensities (LI), and (3) after the external application of β-ionone in both cultures.

**Results:**

The results showed that β-cyclocitral and β-ionone are produced in all growth phases of LTPNA 01 and LTPNA 08. Both strains were producers of β-cyclocitral and β-ionone in normal culture conditions. It was observed that the β-cyclocitral concentration was higher than β-ionone in all light intensities investigated in this study. Additionally, the strain LTPNA 01 produced more β-cyclocitral than LTPNA 08 at almost all times and LIs analyzed. However, the strain LTPNA 08 produced more β-ionone, mainly at the initial times. In addition, the experiment results with the external addition of β-ionone in the cultures showed that the strain LTPNA 01 produced more β-cyclocitral in control conditions than in treatment. Nonetheless, β-ionone production was higher in treatment conditions in LTPNA 08, indicating that the addition of β-ionone may favor the production of these compounds and inhibit the production of β-cyclocitral.

**Conclusion:**

Our results showed that some abiotic factors, such as different light intensities and external application of β-ionone, can be triggers that lead to the production of VOCs.

**Supplementary Information:**

The online version contains supplementary material available at 10.1186/s12866-022-02473-6.

## Background

Cyanobacteria have been widely studied due to the production of secondary metabolites [1–4], which can impact the environment and water quality. Some species can produce cyanotoxins, which pose a risk to human and animal health [5]. In addition, algae and cyanobacteria produce hundreds of volatile organic compounds (VOCs), which comprise odors, taste-altering substances, allelopathic compounds and growth regulators [6–8]. VOCs are often found in eutrophic waters, contributing to the earthy/mold odor and impacting the drinking water supply as consumers complain about the water quality. Thus, increasing water treatment costs [6].

**Fig. 1:**
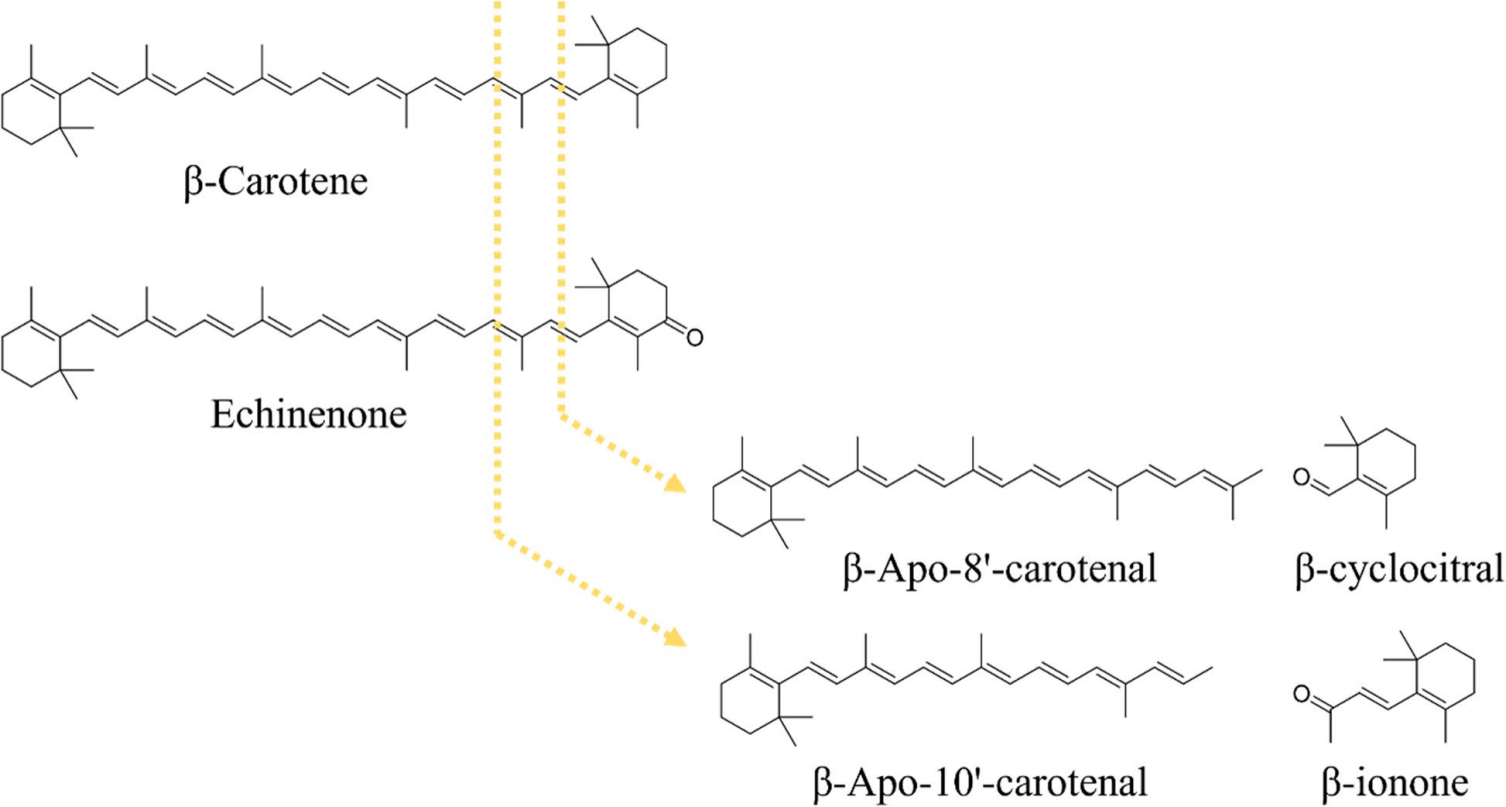
Formation of β-ionone and β-cyclocitral via enzymatic or non-enzymatic oxidative cleavage of the carotenoid β–carotene or echinenone (main carotenoids found in cyanobacteria): yellow arrow indicates oxidation of the double bonds in the carotenoid backbone. Echinenone is converted in hydroxylated analogous variants. Based on Michel Havaux 2013

One of the main VOCs produced by cyanobacteria are β-cyclocitral and β-ionone, from the nor-carotenoid group, resulting from the oxidation of β-carotene (one of the main photosynthetic pigments of cyanobacteria) in the cells of these microorganisms (Fig. 1) [6]. Furthermore, β-cyclocitral and β-ionone are the main metabolites of *Microcystis*, a relevant genus in cyanobacterial blooms [7] and which is also associated with the production of microcystins (hepatotoxins) [9].

The substantial decomposition of cyanobacterial residues can release toxins and high concentrations of β-cyclocitral and β-ionone. These compounds have lytic activity against cyanobacteria, can regulate the growth of cyanobacteria and interfere in the production of volatile compounds [10, 11]. Furthermore, volatile compounds can be affected by environmental factors such as light intensity, temperature, nutritional conditions and abiotic stresses [6, 12, 13]. Furthermore, these compounds have been involved in photooxidative stress tolerance in photosynthetic microorganisms and vascular plants [14].

Studies have described that light intensity stimulates the growth of *Microcystis* and other cyanobacteria and, consequently, interferes with the production of secondary metabolites [15, 16]. Furthermore, a recent study [17] showed that β-cyclocitral production gradually intensified with increasing light and temperature. Thus, it is necessary to understand the triggers that lead to VOC production, as these responses will help us to understand the ecological functions of VOC emissions in nature [18].

Therefore, the general objective of this work was to evaluate the VOCs produced by two strains of *Microcystis aeruginosa* (non-producing and toxin-producing). The analysis of VOC production was carried out in (1) normal culture conditions, (2) under different light intensities (LI), and (3) after the external application of β-ionone in both cultures.

## Methods

### Microcystis strains selection and culture conditions

Cyanobacteria were selected among the strains already isolated and maintained as non-axenic cultures at the Laboratory of Toxins and Natural Algae Products (LTPNA) of the Faculty of Pharmaceutical Sciences, University of São Paulo, FCF-USP. The selection was performed according to their VOC profile, β-ionone, β-cyclocitral, geosmin (1,10-dimethyl-trans-9-decalol), and 2-MIB (2-methylisoborneol). The cyanobacteria strains selected were *Microcystis aeruginosa* LTPNA 08 (microcystin-producing) (NCBI: OL584204) and *Microcystis aeruginosa* LTPNA 01 (microcystin non-producing) (NCBI: OL584203), both strains were identified by 16S rRNA sequencing. They were cultured in ASM-1 (Artificial Seawater Media) culture medium pH 8.0 [19], with aeration, at a temperature of 24 ± 2 °C, and photoperiod of 12 h of light and 12 h of darkness, and the light intensity (LI) were specific for each experiment.

In the normal culture conditions and the β-ionone addition experiment, LI, 50 μmol photons m-^2^ s-^1^ were used. While, in the experiments to evaluate the production of VOCs in different LI, three different LI were used, 50 μmol photons m-^2^ s-^1^, 150 μmol photons m-^2^ s-^1^ and 250 μmol photons m-^2^ s-^1^.

### Growth determination by cell count

Initially, 10 mL of each culture were transferred to 90 mL media ASM-1. The growth of the cell cultures was monitored by cell counting in a Fuchs-Rosenthal hemocytometer (KASVI, Germany) in an optical microscope ZEISS Primo Star (Jena, Germany) for 30 days. Exponential regressions were applied to the variation of the number of cells over the growth time to determine the exponential growth phase. The exponential phase was defined as the period in which the regression showed data adherence of at least 95% or R2 ≥ 0.95. The cell density was expressed as cell mL^−1^ [20, 21]*.*

### VOC production, SPME headspace sampling, and GC–MS analysis

The strains LTPNA 01 and LTPNA 08 were cultured in an ASM-1 culture medium to search for VOCs produced in different stages of cyanobacterial growth. Cultures were maintained for 18 days in the conditions mentioned in the *Cyanobacteria selection and culture conditions* subsection; the collections were performed every 6 days (T6, T12, and T18) and the experiment was carried out in triplicate.

These cultures were subjected to a headspace solid-phase micro-extraction (HS-SPME) (Supelco, Bellefonte, PA, USA), coupled to GC/MS (Agilent technologies 7890A with mass selective detector 5975C) for the quantitative determination of volatile compounds, as previously described by Fujise et al. [22]. Briefly, 20 ml vials with 10 ml of cyanobacteria culture, 4 g of NaCl, and an internal standard solution were added; the vials were sealed and then shaken to dissolve the salt. The flasks were subsequently incubated and placed on a shaker equipped with a heating block for 5 min at 60 ºC. An SPME external needle of 50/30 μm divinylbenzene/carboxen/polydimethylsiloxane (DVB/CAR/PDMS) fiber (Supelco, Bellefonte, PA, USA) was passed through the septum and the fiber extended into the upper space. After 20 min, the SPME fiber was subsequently inserted into the flask through the septum, exposed to the gas phase for 30 min and taken to a GC–MS for desorption of the analytes (desorption time = 4 min), separation, and detection. The analyzes conditions of the fiber choice and exposure time, the sample’s agitation time, temperature, among other variables, were standardized according to the methodologies described in the literature [10, 23, 24].

An Agilent 7890A technologies GC–MS with a 5975C selective mass detector, an HP-5MS column (30 m × 0.25 mm ID × 0.25 μm film; Agilent Technologies, USA), and a split-splitless injector were used. The injector temperature was maintained at 250 ºC, and the detector temperature at 280 ºC. The temperature was programmed to stay at 60 ºC for 5 min, followed by a 10 ºC.min^−1^ increase until 120 ºC, and then a 40 ºC.min^−1^ increase until 280 ºC. The MS scanning spectrum was adjusted between the m/z 45 and 250. The ions were simultaneously monitored in SCAN mode.

For identifying and confirming the VOCs produced by the strains, the β-cyclocitral and β-ionone standards were obtained from Sigma-Aldrich (São Paulo, Brazil). The identification of the compounds was based on their retention time and mass spectrum profile by GC–MS compared to the analytical standards and the NIST library (NIST, Washington-DC, USA). The data (mg L^−1^) were normalized by cell density (cell mL^−1^) and expressed as μg cell^−1^.

### Evaluation of the effect of different light intensities on VOC production

Three different light intensities (LI) (50, 150, and 250 μmol photons m^−2^ s ^−1^) were tested to evaluate the possible effects on the VOC production profile in both strains, LTPNA 01 and LTPNA 08. The strains were inoculated in 1,700 mL of ASM-1 culture medium with aeration in flasks of 3 L, at a temperature of 24 ± 2° C, under different LI and maintained for 18 days, with sampling every six days. The production of VOCs was analyzed by gas chromatography coupled to mass spectrometry (GC–MS). The data (mg L^−1^) were normalized by cell density (cell mL^−1^) and expressed as μg cell^−1^.

### VOC production in cultures with the addition of β-ionone standard

A β-ionone standard was added to the cultures of the LTPNA 01 and LTPNA 08 strains to assess whether an exogenous volatile compound would influence the growth and production of endogenous volatile compounds (β-ionone and β-cyclocitral) by cyanobacteria. Cultures were performed with 10^5^ mL^−1^ cells in triplicate, three replicated cultures of each strain (biological replicates), and three replicated samples of each culture (technical replicates). The experiment was separated into positive control (without the addition of β-ionone), negative control (addition of 0.01 mM of the standard β-ionone and without strain) and treatment (addition of 0.01 mM of the standard β-ionone) [10, 21]. The cultures were maintained at 24 ºC, under irradiance of 50 μmol photons m^−2^ s^−1^ for 18 days, and sampling was performed every three days. The production of VOCs was analyzed by GC–MS. The data (mg L^−1^) were normalized by cell density (cell mL^−1^) and expressed as μg cell^−1^.

### Statistical analysis

Statistical tests and graphs were performed using Sigmaplot® 11.0 software. The normality of the data was tested using the Shapiro–Wilk test. In the experiments with at least two independent variables that were approved by the normality and equal variance tests, a Two-Way Analysis of Variance (Two-Way ANOVA) was performed, followed by the Tukey post-test for multiple comparisons, to understand how each data group affects the other groups among the variables tested. Additionally, One Way ANOVA was performed, followed by the Tukey post-test, to check the influence of time on the production of VOCs (or cell density) of each strain (and LI or treatment). In addition, a Student t-test (or One Way ANOVA, in the case of *M. aeruginosa* cell growth in different LI) was performed to verify the difference between the groups (strains, LI, or treatment/control) at each moment. When the data failed the normality or equal variance tests and presented at least three groups, a Kruskal–Wallis One Way Analysis of Variance on Ranks was performed, followed by Tukey’s post-test; for comparisons between only two groups, a Mann–Whitney Rank Sum Test was applied. The descriptive level (*p*-value) was used as the primary parameter evaluated to interpret the test results. The level of significance (probability of type I error) adopted for the tests was 5% (0.05).

## Results

### VOC production by Microcystis aeruginosa strains

**Fig. 2:**
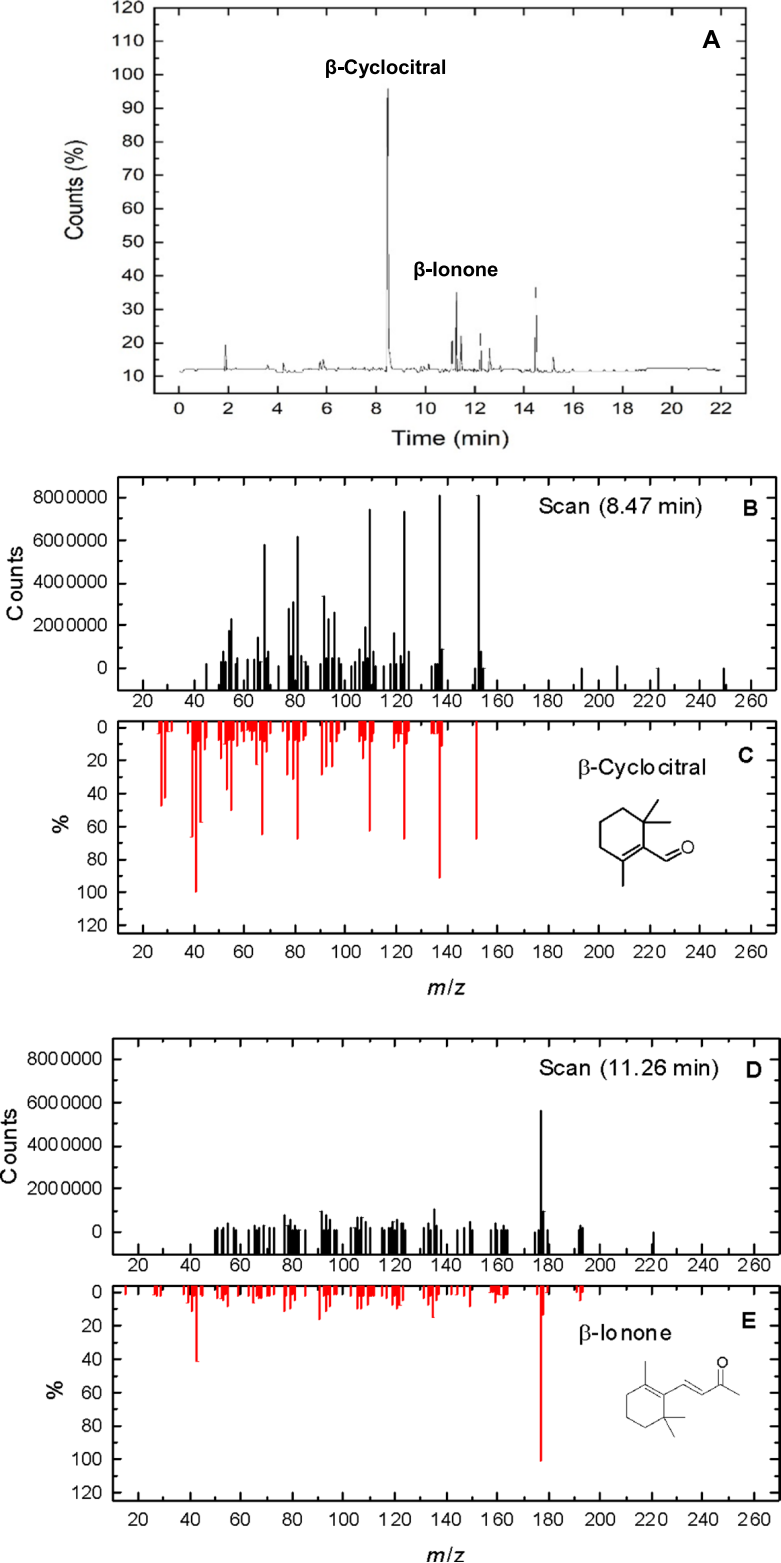
**A** Chromatograms obtained by GC–MS SCAN mode, indicating the peaks of the volatile compounds β-cyclocitral (8.47 min) and β-ionone (11.26 min). **B** Ions extraction at the retention time of 8.47 min. **C** Mass spectra for β-cyclocitral available in the NIST library. **D** Ions extraction at the retention time of 11.26 min. **E** Mass spectra for β-ionone available in the NIST library

During the exponential growth phase of the two *M. aeruginosa* strains (microcystin non-producing LTPNA 01 and microcystin-producing LTPNA 08), VOCs were evaluated. However, only two compounds were detected, β-cyclocitral and β-ionone. In the exponential growth phase, cells are in high metabolic activity aimed at cell division. The compounds were confirmed after comparing the mass spectra monitored in SCAN mode (*m/z* 123, 137, and 152 ions for β-cyclocitral, and *m/z* 105, 149, and 177 ions for β-ionone) with the internal commercial standard of each compound, and also with the data available in the NIST library (National Institute of Standards and Technology, MD, USA) (Fig. 2).

**Fig. 3:**
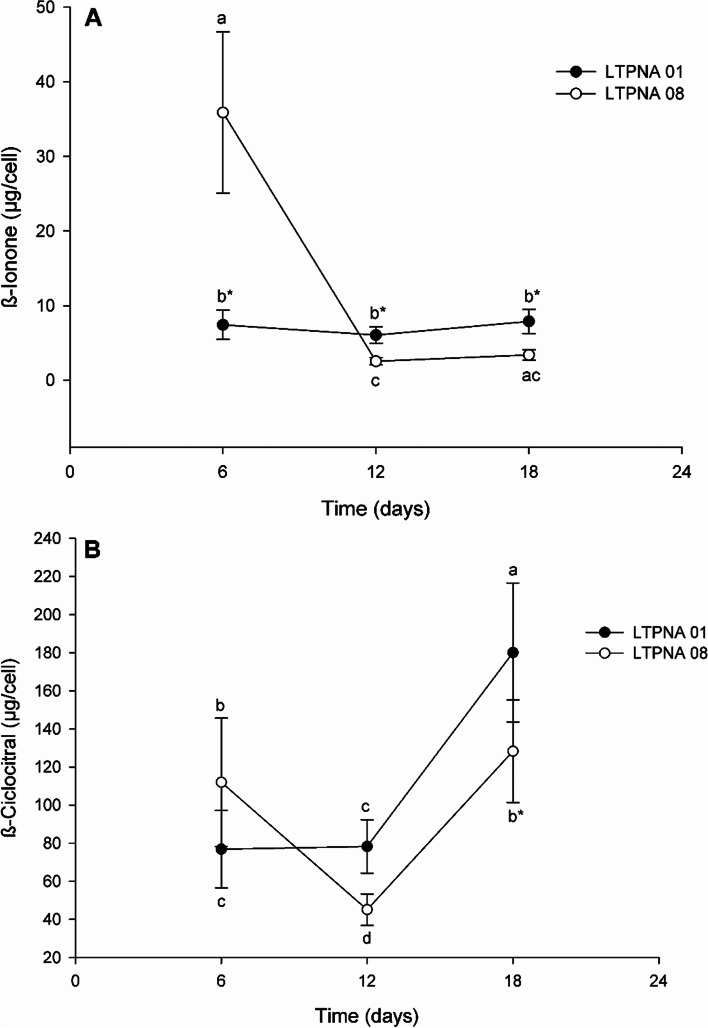
Production (µg cell^−1^) and comparison of β-ionone (**A**) and β-cyclocitral (**B**) compounds during different growth times in toxin-producing (LTPNA 08) and non-producing (LTPNA 01) strains of *M. aeruginosa*. Different letters represent significant differences in overgrowth time in each strain (for β-ionone in LTPNA 08: Kruskal–Wallis One Way Analysis of Variance on Ranks followed by Tukey post-test, *p* < 0.05; for β-ionone in LTPNA 01: One Way Analysis of Variance followed by Tukey post-test, *p* < 0.05; and for β-cyclocitral: Two Way Analysis of Variance followed by Tukey post-test, *p* < 0.05). * Represents a significant difference between the strains for each growth time (Student t-test, *p* < 0.05)

The production of compounds β-cyclocitral and β-ionone were observed at all times of the exponential growth phase in both LTPNA 01 and LTPNA 08 (Fig. 3) strains. The production of β-ionone (Fig. 3A) was higher (*p* = 0.011) in the strain LTPNA 01 only at the initial time (T6) while for the other times, the profile observed was the inverse; LTPNA 08 produced more β-ionone than LTPNA 01 at T12 (*p* = 0.007) and T18 (*p* = 0.011). However, the production of β-ionone in the strain LTPNA 01 did not change with the time (*p* = 0.404), while for LTPNA 08, there was a significant reduction from T6 to T12 (*p* = 0.011), both showing no difference concerning T18.

The β-cyclocitral compound (Fig. 3B) was detected in higher abundance than β-ionone in both strains. The production of β-cyclocitral was higher (*p* = 0.028) in the strain LTPNA 01 only at the final time of the exponential phase (T18), while for the other times analyzed, there was no difference between LTPNA 08 and LTPNA 01 (T6, *p* = 0.116 and T12, *p* = 0.135). The production of β-cyclocitral in the strain LTPNA 01 was maintained in the initial times T6 and T12 (*p* = 0.997) but increased at T18 (*p* = 0.001). In contrast, for the strain LTPNA 08, there was a significant reduction from T6 to T12 (*p* = 0.019), followed by an increase in T18 (*p* = 0.005), reaching the same concentration as in T6 (*p* = 0.718).

### Influence of different light intensities on VOC production

The strain LTPNA 01 grew exponentially until the 18^th^ day of cultivation in the LIs 50 and 150 µmol photons m^−2^ s^−1^; the LI 250 µmol photons m^−2^ s^−1^ started to present cell death after the time T6 (Figure S1A). The strain LTPNA 08 also grew exponentially until the 18^th^ day of cultivation in the three LI analyzed (Figure S1B). According to Figure S1A, there are no statistical differences between the LI 50 and 150 µmol photons m^−2^ s^−1^, and 150 and 250 µmol photons m^−2^ s^−1^, while the cell density was higher in the LI 50 than in 250 µmol photons m^−2^ s^−1^ (*p* = 0.004) at T0 in LTPNA 01. The cell density was significantly different between the LI in the times T6 and T18 (*p* < 0.001), i.e., cell density decreased with the elevation of the LI. Likewise, the profile observed at T12 was similar to T0, and there were no differences between the LIs 50 and 150 µmol photons m^−2^ s^−1^, and 150 and 250 µmol photons m^−2^ s^−1^, and a higher cell density in the LI 50 µmol photons m^−2^ s^−1^ compared to the LI 250 µmol photons m^−2^ s^−1^ (*p* = 0.004).

The LTPNA 08 strain (Figure S1B) showed cell densities significantly different between the LI in the times T0, T6, and T12 (*p* < 0.001). However, the cell density profile (which was increasing with the LIs, in T0) changed in the other times (decreasing with the elevation of the LI). Additionally, no differences were observed between the LI 50 and 150 µmol photons m^−2^ s^−1^, and 150 and 250 µmol photons m^−2^ s^−1^, while the cell density was higher in the LI 50 than in 250 µmol photons m^−2^ s^−1^ (*p* = 0.004) at T18.

**Fig. 4:**
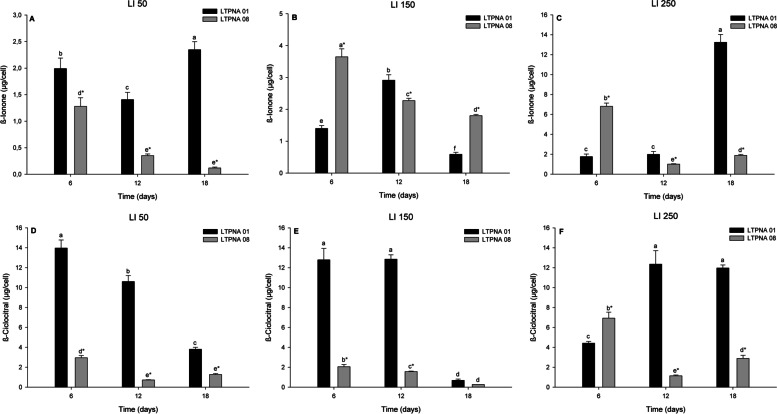
Production of volatile compounds (β-ionone and β-cyclocitral) during the growth times of toxin-producing (LTPNA 08) and non-producing (LTPNA 01) strains of *M. aeruginosa* under different light intensities (LI). **A** LI 50 µmol photons m^−2^ s^−1^, **B** LI 150 µmol photons m^−2^ s^−1^, and **C** LI 250 µmol photons m^−2^ s^−1^ for β-ionone. **D** LI 50 µmol photons m^−2^ s^−1^, **E** LI 150 µmol photons m^−2^ s^−1^, and **F** LI 250 µmol photons m^−2^ s^−1^ for β-cyclocitral. Different letters represent significant differences over the growth time in each strain and LI (for β-ionone in LI 50 and LI 150, and β-cyclocitral in LI 50 and LI 250: Two Way Analysis of Variance followed by Tukey post-test, *p* < 0.05; for β-ionone in LI 250 and β-cyclocitral in LI 150: One Way Analysis of Variance followed by Tukey post-test, *p* < 0.05). * Represents a significant difference between the strains for each growth time (Student t-test or Mann–Whitney Rank Sum Test (at 18 days for β-cyclocitral in LI 150), *p* < 0.05)

The compounds β-cyclocitral and β-ionone were produced during the exponential growth phase of both strains (LTPNA 01 and LTPNA 08) in the LI 50, 150, and 250 µmol photons m^−2^ s^−1^ (Fig. 4). In addition, β-cyclocitral was higher than β-ionone at almost all times and LIs investigated.

Analyzing β-ionone production, the strain LTPNA 01 showed a higher concentration of this compound (Figs. 4A, 4B and 4C) than the strain LTPNA 08 in all the growth times (*p* < 0.001) in the LI 50 µmol photons m^−2^ s^−1^, reducing the production from T6 to T12 (*p* < 0.001) and then an increase from T12 to T18 (*p* < 0.001). Likewise, The LTPNA 08 also showed a reduction from T6 to T12 (*p* < 0.001) in the LI 50 µmol photons m^−2^ s^−1^, but then the production remained constant until T18 (*p* = 0.13). The production of β-ionone was also significantly different between the strains; at T6 and T18, its concentration was higher in the strain LTPNA 08, while in T12, the inverse was observed (higher in the strain LTPNA 01) in the LI 150 µmol photons m^−2^ s^−1^. The LTPNA 01 showed an increase in β-ionone production from T6 to T12 (*p* < 0.001), followed by a decrease in T18 (*p* < 0.001), while the strain LTPNA 08 showed a decrease in the production of this VOC with time (*p* ≤ 0.003). Analyzing LI 250 µmol photons m-2 s^−1^, the production of β-ionone was also significantly different between the strains; at T6, its concentration was higher in the strain LTPNA 08, but at T12 and T18, the inverse was observed (higher in the strain LTPNA 01). The production of β-ionone in the strain LTPNA 01 remained constant from T6 to T12 (*p* = 0.853) and increased in T18 (*p* =  < 0.001), while in the strain LTPNA 08 it decreased from T6 to T12 (*p* < 0.001) and increased again in T18 (*p* = 0.004).

According to β-cyclocitral production (Figs. 4D, 4E, and 4F), the strain LTPNA 01 showed a higher concentration of this compound compared to LTPNA 08 in almost all the times and LI studied, except T18 in the LI 150 µmol photons m^−2^ s^−1^ (*p* = 0.100) and T6 in the LI 250 µmol photons m^−2^ s^−1^ (*p* < 0.001). In the lowest LI, 50 µmol photons m^−2^ s^−1^, the β-cyclocitral production in the strain LTPNA 01 reduced with the time (*p* < 0.001), while in the strain LTPNA 08, there was a reduction from T6 to T12 (*p* < 0.001) and the production remained constant until T18 (*p* = 0.304). At the LI 150 µmol photons m^−2^ s^−1^, the production of this compound in the strain LTPNA 01 remained constant from T6 to T12 (*p* = 0.993) and showed a significant decrease in T18 (*p* < 0.001), while in the strain LTPNA 08, the concentration reduced with the time (*p* ≤ 0.009). The β-cyclocitral production in the strain LTPNA 01 in the LI 250 µmol photons m^−2^ s^−1^ increased from T6 to T12 (*p* < 0.001) and showed no difference from T12 to T18 (*p* = 0.739), while in the strain LTPNA 08, the concentration in all times was significantly different, decreasing from T6 to T12 (*p* < 0.001) and increasing again from T12 to T18 (*p* = 0.014).

### Influence of the addition of β-ionone standard on VOC production

Cell growth curves (Figure S2) were performed in control (without the standard addition), and treatment conditions (with the addition of the β-ionone standard) and the data of cell density (cell mL^−1^) was used to normalize the production of VOCs (mg L^−1^), which was then expressed as μg cell^−1^.

The strains LTPNA 01 and LTPNA 08 grew exponentially until the 18^th^ day of cultivation (Figure S2). In the strain LTPNA 01 (Figure S2A) no statistical differences were observed between the control and treatment conditions for T0 (*p* = 0.165) and T6 (*p* = 0.200), while these two conditions were significantly different in T12 (*p* = 0.008) and T18 (*p* ≤ 0.001). In the control condition, LTPNA 01, T18 was significantly higher than T0 (*p* = 0.016), but the cell density in these times was not different from the other times, while treatment conditions showed statistical difference in all the times, increasing according to the time (*p* < 0.001). In the strain LTPNA 08 (Figure S2B) no statistical differences were observed between the control and treatment conditions for the times T6 (*p* = 0.090) and T12 (*p* = 0,121), while these two conditions were significantly different in T0 (*p* = 0.026) and T18 (*p* = 0.025). For the strain LTPNA 08, the cellular growth profile was similar to the strain LTPNA 01. In the control condition, T18 was significantly higher than T0 (*p* = 0.016), but the cell density in these times was not different from the other times, while the treatment condition presented statistical difference in all the times, increasing according to the time (*p* ≤ 0.001).

**Fig. 5:**
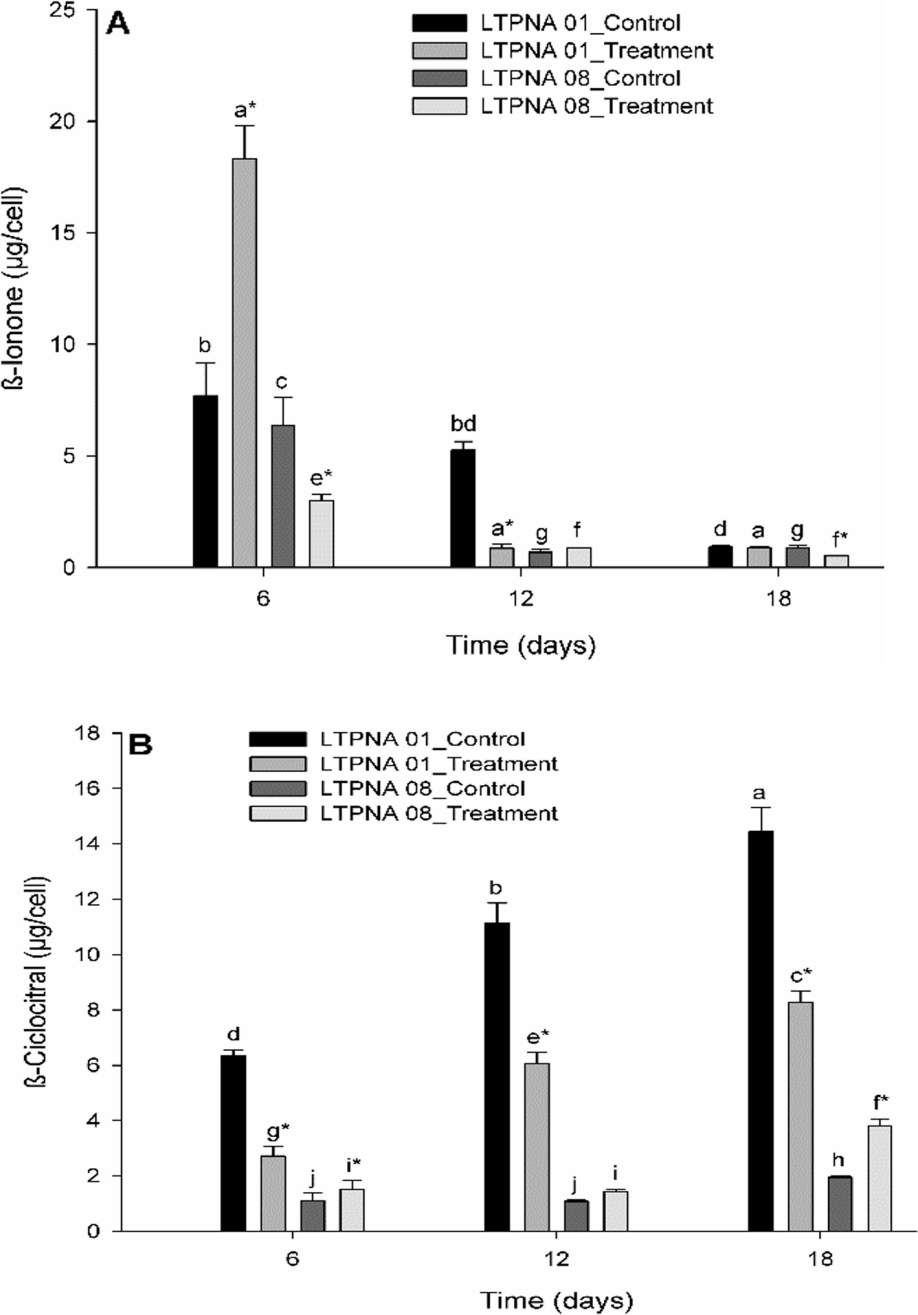
Effects of the addition of β-ionone standard in the production of volatile compounds (**A** β-ionone and **B** β-cyclocitral) during the growth times of toxin-producing (LTPNA 08) and non-producing (LTPNA 01) strains of *M. aeruginosa*. Different letters represent significant differences over the growth time in each strain and condition, control (without the addition of β-ionone) or treatment (with the addition of β-ionone) (for β-ionone in LTPNA 01: Kruskal–Wallis One Way Analysis of Variance on Ranks followed by Tukey post-test, *p* < 0.05; for β-ionone in LTPNA 08: One Way Analysis of Variance followed by Tukey post-test, *p* < 0.05; and for β-cyclocitral: Two Way Analysis of Variance followed by Tukey post-test, *p* < 0.05). * Represents a significant difference between the conditions (control and treatment) for each growth time (Student t-test, *p* < 0.05)

As expected, it was possible to detect β-ionone and β-cyclocitral in both strains (Fig. 5). The β-ionone production (Fig. 5A) was statistically different between the control and treatment conditions of the strain LTPNA 01 in the times T6 (*p* ≤ 0.001, higher in the treatment) and T12 (*p* ≤ 0.001, higher in control), but not different in T18 (*p* = 0.627). In the LTPNA 01 control condition, the compound concentration presented no statistical difference between T6 and T12 and T12 and T18, but there was a significant decrease from T6 to T18 (*p* = 0.004). For the LTPNA 01 treatment condition, there was no statistical difference between the growth times (*p* = 0.050). For the strain LTPNA 08 (Fig. 5B), the β-ionone production was statistically different between the control and treatment conditions in the times T6 (*p* = 0.011) and T18 (*p* = 0.005), but not different in T12 (*p* = 0.101). In the LTPNA 08 control condition, the compound concentration presented a significant reduction from T6 to T12 (*p* < 0.001), and then remained constant until T18 (*p* = 0.943). The β-ionone production in the LTPNA 08 treatment condition presented a similar profile from the control condition, reducing from T6 to T12 (*p* < 0.001) and then remaining constant until T18 (*p* = 0.081). Thus, these results show that the addition of exogenous β-ionone did not influence the production of β-ionone in some growth times.

The β-cyclocitral production was statistically different at all times analyzed for the strain LTPNA 01, the control and treatment conditions (*p* < 0.001), with increasing production of the compound over time (Fig. 5B). Additionally, the production of β-cyclocitral by LTPNA 01 was significantly higher in the control condition at all times analyzed (*p* < 0.001), and at T18 the highest production of this metabolite was observed. The profile of β-cyclocitral production in the strain LTPNA 08 remained constant from T6 to T12 (*p* = 0.997 for the control and *p* = 0.835 for the treatment) and increased in T18 (*p* < 0.001). These results showed that the addition of β-ionone negatively influenced the production of β-cyclocitral in the LTPNA 01 strain.

The results showed that the production pattern of the β-ionone and β-cyclocitral were higher in the LTPNA 01 strain compared to the LTPNA 08 strain. In general, the production of β-cyclocitral was higher than β-ionone.

## Discussion

*Microcystis* species are the most common bloom cyanobacteria in several countries (26). Despite extensive studies on the production of bioactive cyanopeptides in this genus, there are limited data on strains isolated from Brazil [25–27].

In this work, the production of VOCs (β-cyclocitral and β-ionone) was analyzed and compared over the growth time of the LTPNA 01 and LTPNA 08 strains of *M. aeruginosa*. It was noted that the production of these compounds was more intense in the non-producing strain (LTPNA 01) than the toxin-producing strain (LTPNA 08), specifically in this study. The high concentration of the β-cyclocitral compound in the LTPNA 01 and LTPNA 08 strains at the different growth times studied may be due to the greater β-carotene biosynthesis performed by *Microcystis*. According to several studies, the compounds β-cyclocitral and β-ionone are cyclic terpenes generated from the oxidative decomposition of β-carotene and are largely produced by species of *Microcystis* [28–31]. Also, Pabby et al. [32] showed that the highest concentrations of these compounds are observed in cells in the exponential growth phase compared to cells in the stationary growth phase. Additionally, corroborating with our studies, [25] it was observed that the concentrations of β-cyclocitral were relevant for the growth phases of *Microcystis*.

In addition, the influence of different light intensities on the growth and VOC production in both cyanobacteria strains (LTPNA 01 and LTPNA 08) analyzed in this study corroborate a previous study [28], which reported that under conditions of high luminosity (300 μmol m^−2^ s^−1^), a low growth rate was detected in *M. aeruginosa* CPCC632 and *Synechocystis* sp. FACHB898.

According to Zheng et al. [17], cell growth inhibition may be explained by the accumulation of reactive oxygen species (ROS) under stress conditions, such as high light and temperature, pH, and salinity, among other parameters. Furthermore, Zheng et al. [17] showed that ROS levels in *M. aeruginosa* cells increased with increasing LI, and ROS accumulation resulted in oxidative damage, causing cell growth inhibition. In addition, as in the study by Sigaud-Kutner et al. [29] it was observed that the content of β-carotene and lycopene did not vary significantly with the light–dark cycle, as well as in high light and moderate light combined with other stressful conditions, such as depletion of nutrients, which reduce the photosynthetic rate of *M. aeruginosa* and increase the degree to which the absorbed light can be excessive [33, 34].

The compounds β-cyclocitral and β-ionone were produced by both strains in all LI analyzed and β-cyclocitral was higher than β-ionone in almost all times and LI investigated, mainly by LTPNA 01. As in our study, stress caused by high light intensity was also reported [17, 35]. However, they showed an increased production of β-cyclocitral in higher LI, i.e., unfavorable environmental conditions (such as high light intensity or drought) positively influence *Microcystis* growth rates and increase the production of volatile compounds. In addition, Cordara et al. [36] reported that cyanobacteria from the genus *Synechocystis* showed a significant growth and photosynthetic activity with reduced light intensity up to 500 µmol photons m^−2^ s^−1^ and entered the photoinhibition state only at 800 μmol photons m^−2^ s^−1^.

Bittencourt-Oliveira et al. [37] presented similar results when exposing strains of *Cylindrospermopsis raciborskii* to different LI. Both strains studied increased their growth rates when subjected to a higher LI. However, studies such as Huisman et al. [38] described that cyanobacteria species are tolerant to high light irradiations, due to the increased production of carotenoids and photoprotective pigments, which optimize photosynthetic efficiency, and are persistent in water samples. Thus, this study differs from others that found that higher irradiations positively influence growth rates and production of volatile compounds in some cyanobacteria species.

**Fig. 6:**
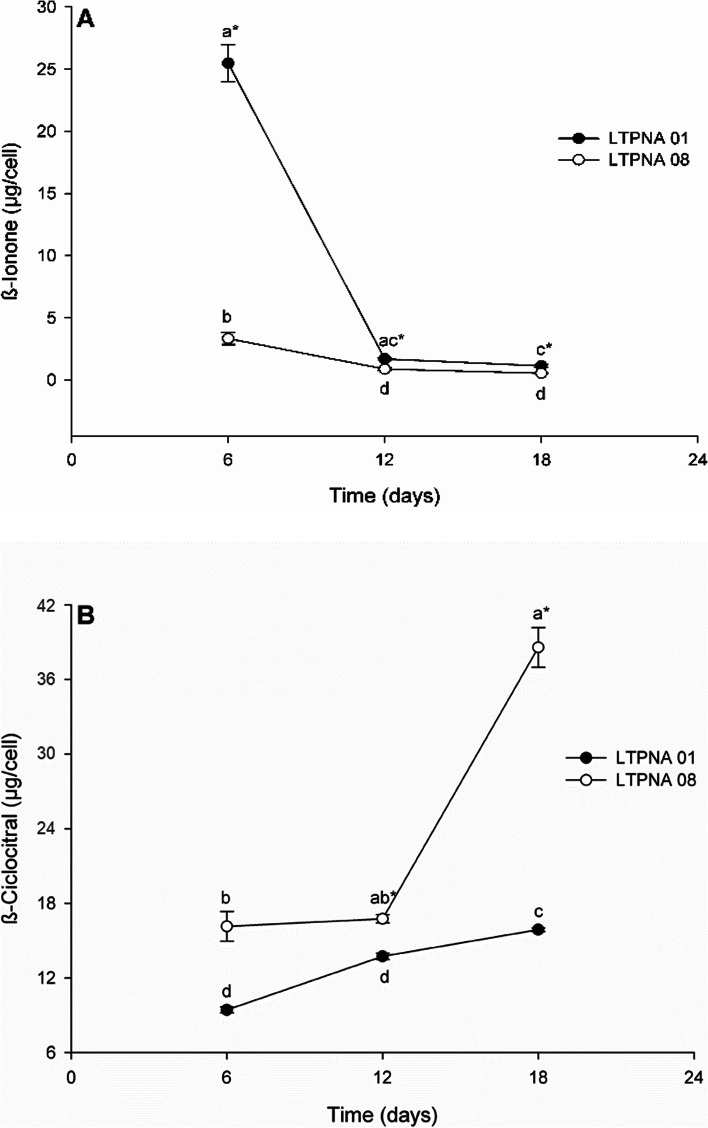
Comparison of VOCs production by M. aeruginosa LTPNA 01 and LTPNA 08 during growth time in the treatment (addition of β-ionone standard). A. β-ionone production by both strains. B. β-cyclocitral production by both strains. Different letters represent significant differences overgrowth time in each strain (for both compounds in LTPNA 01: Kruskal–Wallis One Way Analysis of Variance on Ranks followed by Tukey post-test, *p* < 0.05; for both compounds in LTPNA 08: One Way Analysis of Variance followed by Tukey post-test, *p* < 0.05). * represents a significant difference between the strains for each growth time (Student t test or Mann–Whitney Rank Sum Test (at 6 days for β-cyclocitral), *p* < 0.05)

β-ionone can regulate the cyanobacteria growth and interfere in the production of volatile compounds [10], to assess whether there was an inhibition or increase in cell growth and production of volatile compounds in our study, 0.01 mM of β-ionone was added to the cultures (Fig. 5 and Fig. 6). However, specifically in this study, which compares two strains of *Microcystis*, the non-producing strain showed a higher VOC production in control conditions. Although further studies are needed, this is the first report correlating the negative influence of β-ionone on β-cyclocitral biosynthesis in a non-toxin-producing strain of *M. aeruginosa*. Thus, there seems to be no relationship between the production of these compounds and the production of toxins. Nonetheless, in the work of Chen et al. [33], the authors found, for the first time, significant correlations between the concentrations of β-cyclocitral and β-ionone and the concentrations of microcystin. In contrast, Shao et al. [34] explored the potential toxicity targets of β-ionone in the *M. aeruginosa* photosynthetic system. β-ionone stress led to a decrease in the cells’ pigment content and the cells’ thylakoid membrane began to collapse when *M. aeruginosa* was exposed to β-ionone, demonstrating how sensitive the cells were to the β-ionone addition.

## Conclusions

In conclusion, VOCs, such as β-cyclocitral and β-ionone, have gained increasing attention in recent years as they are identified as some of the primary contaminants of drinking water, negatively impacting its taste and odor. In this sense, β-cyclocitral and β-ionone were produced by the cyanobacteria *Microcystis aeruginosa* strains LTPNA 01 and LTPNA 08. The concentrations of β-cyclocitral produced by both *Microcystis* strains were higher than the concentrations of β-ionone. Additionally, the lowest light intensity, 50 μmol photons m^−2^ s^−1^, favored the growth of both strains; and VOC production varied according to the LI and time studied.

Furthermore, the addition of the volatile compound β-ionone to the cultures negatively influenced the production of the compound β-cyclocitral. Thus, it can be inferred that VOC production can undergo qualitative and quantitative changes depending on the environmental stimulus present, mainly due to abiotic factors (such as the addition of β-ionone and light intensity). Overall, these results are promising but demonstrate a fundamental need for more focused research on the chemical ecology of VOCs in aquatic ecosystems.

## Supplementary Information

**Additional file 1. **

**Additional file 2. **

## Data Availability

The datasets generated and analyzed during the current study are partially available in the SIMONE VIEIRA repository, https://www.teses.usp.br/teses/disponiveis/9/9141/tde-12122017-124445/pt-br.php.

## Abbreviations

VOCs: Volatile Organic Compounds
ASM: Artificial Seawater Media
LTPNA: Laboratory of Toxins and Natural Algae Products
FCF-USP: Faculty of Pharmaceutical Sciences, University of São Paulo
2-MIB: 2-Methylisoborneol
HS-SPME: Headspace Solid-Phase Micro-Extraction
GC–MS: Gas Chromatograph Coupled to a Mass Spectrometer
DVB/CAR/PDMS: Divinylbenzene/Carboxen/Polydimethylsiloxane
LI: Light intensity
ROS: Reactive Oxygen Species
