# Supplementary material for: Effects of different cultivation conditions on the production of β-cyclocitral and β-ionone in Microcystis aeruginosa
Source: BMC Microbiol. 2022 Mar 24;22:78. doi: 10.1186/s12866-022-02473-6 (PMC8944028; doi:10.1186/s12866-022-02473-6)
Supplement: Supplementary file 2 — Additional file 2. [file 12866_2022_2473_MOESM2_ESM.docx]

**"Effects of different cultivation conditions on the production of β-cyclocitral and β-ionone in *Microcystis aeruginosa*".**

Jéssica Aparecida Silva Moretto^1*^, Paloma Nathane Nunes de Freitas^1,3^, Éryka Costa de Almeida^2^, Lucas Miguel Altarugio^3^, Simone Vieira da Silva^2^, Marli de Fátima Fiore^1^, Ernani Pinto^1,2,4*^

*^1^ Centre for Nuclear Energy in Agriculture, University of São Paulo, Piracicaba, SP, Brazil.*

*^2^ Faculty of Pharmaceutical Sciences, University of São Paulo, São Paulo, SP, Brazil.*

*^3^ Luiz de Queiroz College of Agriculture, University of São Paulo, Piracicaba, SP, Brazil.*

*^4^ Food Research Center (FoRC – CEPID), University of São Paulo, São Paulo, SP, Brazil.*

^*Corresponding author: jessica.moretto@usp.br (Moretto, J.A.S.) and^ [^ernani@usp.br^](mailto:ernani@usp.br) ^(Pinto,E)^

Figure S1. Growth curves of non-producing (**A**. LTPNA 01) and toxin-producing (**B**. LTPNA 08) strains of *M. aeruginosa* grown under different light intensities (LI 50, 150, and 250 μmol photons m^-2^s^-1^). Different letters represent significant differences over the growth time in each LI and strain (One Way Analysis of Variance followed by Tukey post-test, p < 0.05). * represents a significant difference between the LIs for each growth time (One Way Analysis of Variance followed by Tukey post-test, p < 0.05 or Kruskal-Wallis One Way Analysis of Variance on Ranks followed by Tukey post-test (for 0 and 12 days of growth time in LTPNA 01, and for 18 days in LTPNA 08), p < 0.05).







Figure S2. Growth curves of non-producing (**A**. LTPNA 01) and toxin-producing (**B**. LTPNA 08) strains of *M. aeruginosa* under different conditions, Control (without addition of β-ionone standard) and Treatment (with addition of β-ionone standard). Different letters represent significant differences over the growth time in each strain and condition, control (without the addition of β-ionone) or treatment (with the addition of β-ionone) (for LTPNA 01 and LTPNA 08 (both in Control): Kruskal-Wallis One Way Analysis of Variance on Ranks followed by Tukey post-test, p < 0.05; for LTPNA 01 and LTPNA 08 (both in Treatment): One Way Analysis of Variance followed by Tukey post-test, p < 0.05). * represents a significant difference between the conditions (control and treatment) for each growth time (Student t test, p < 0.05 or Kruskal-Wallis One Way Analysis of Variance on Ranks followed by Tukey post-test (for 6 days of growth time in LTPNA 01), p < 0.05).
